# Feasibility Assessment of Autologous Human Immune System (HIS) ImmunoGraft Platform Development Using Autologous Mobilized Peripheral Blood (MPB) CD34 Cells Derived from Adult HNSCC Patient

**DOI:** 10.3390/ijms26115269

**Published:** 2025-05-30

**Authors:** Bhavna Verma, Georgia Zhuo Chen, Edmund K. Waller, Mihir Patel, Allyson Anderson, Neal Goodwin, Amy Wesa, Yong Teng, Nabil F. Saba

**Affiliations:** 1 Champions Oncology, Inc., Rockville, MD 20850, USA; ursbhavna@gmail.com (B.V.); amywesa@gmail.com (A.W.); 2 Winship Cancer Institute, Emory University, Atlanta, GA 20850, USA; gzchen@emory.edu (G.Z.C.); ewaller@emory.edu (E.K.W.); mihir.r.patel@emory.edu (M.P.); anderson.allyson@emory.edu (A.A.); yong.teng@emory.edu (Y.T.); 3 Department of Otolaryngology, Emory University, Atlanta, GA 20850, USA; 4 Department of Hematology and Medical Oncology, Emory University, Atlanta, GA 20850, USA

**Keywords:** immunograft, autologous stem cells, PDX, autologous xenograft

## Abstract

Humanized mice generated by hematopoietic stem cell (HSC) transplantation are limited by the immune system developed being allogeneic to the tumor. We have innovated a platform to reconstitute an autologous human immune system (HIS) in immunodeficient NOG-EXL mice from mobilized peripheral blood (MPB)-CD34 cells, along with PDX generated from the same patient’s tumor tissue. Patients consented under an IRB-approved protocol for tumor biopsy and HSC apheresis at Emory University. HSC collection included mobilization with G-CSF and plerixafor, immunomagnetic bead isolation with CliniMACS, and cryopreservation of CD34^+^ cells. PDX were established from biopsies or surgical specimens by passaging into immunodeficient mice. Irradiated NOG-EXL mice were engrafted with HSCs by intravenous transplantation of CD34^+^ HSC. Engraftment of human T cells, B cells, and myeloid cells in peripheral blood was assessed by serial flow cytometry of blood samples, with final assessment of immune components in spleen and bone marrow at 30 weeks. Twenty-eight PDX models were generated from 43 patients with HNSCC; 1 patient underwent apheresis. HSC engraftment in blood was observed in 100% of NOG-EXL mice at 8 weeks post-transplant, with 5–20% hCD45^+^ cells present in the periphery. B-cell development was predominant at early time points and declined over time. Human T-cell and subset development of CD4^+^ and CD8^+^ T cells were observed in blood from 15 weeks post-transplant. Strong development of the myeloid lineage (CD33^+^) was observed starting at 8 weeks and persisted throughout the study. These data demonstrate that mobilization and apheresis of HNSCC patients is technically and clinically feasible and may allow the establishment of autologous HIS-PDX mice.

## 1. Introduction

The majority of patients with head and neck squamous cell carcinoma (HNSCC) are diagnosed with locally advanced regional disease, and in more than 50 percent of cases, the disease is incurable and relapses locally or at distant sites [1]. Patients with relapsed or metastatic HNSCC have a poor prognosis and limited therapeutic options. Although immune checkpoint inhibitors (ICIs) have gained momentum in the management of advanced HNSCC, they remain beneficial to a rather limited number of patients [2,3]. Recent evidence has confirmed the immune-suppressive nature of HNSCC through multiple factors, including the suppression of T-cell activity through immune checkpoint pathways, the recruitment of T-regulatory cells, which are naturally immune suppressive, in addition to a tumor microenvironment (TME), often characterized by hypoxia, which further dampens the immune response [4,5]. These characteristics have promoted interest in the development of novel models that reflect these characteristics and maintain the complex intricacies of the patient’s own immune microenvironment. A deeper understanding of the reasons behind the lack of response to ICIs by exploring the activity of novel agents in a reliable pre-clinical model is very attractive.

Patient-derived xenograft (PDX) models that maintain the molecular, genetic, and histological heterogeneity of the original tumor tissues in immune-deficient mice have been successfully developed and have played a substantial role in the evaluation of novel anti-tumor agents. Anti-cancer agents tested in this model include chemotherapy, small molecules, biologics, as well as anti-angiogenics. Recent evidence shows the accuracy of these models in predicting clinical response. However, PDX models have fallen short of fulfilling the need for models that reflect patient and tumor characteristics [6,7].

Cord-blood-based donor-derived human immune system (HIS) models and self-derived adult peripheral blood methods represent two distinct strategies for generating humanized immune models. Each of these has unique advantages and limitations. Cord-blood-derived HIS models typically rely on transplantation of hematopoietic stem cells (HSCs) from allogeneic cord blood into immunodeficient mice, leading to multilineage hematopoiesis and de novo immune system development, including T-cell education in the thymus. This approach enables robust, systemic human immune reconstitution but often exhibits donor-to-donor variability and delayed immune maturation. In contrast, self-derived peripheral blood methods utilize mature immune cells, such as peripheral blood mononuclear cells (PBMCs), from the same individual, offering a rapid and autologous model ideal for studying personalized immune responses, such as those in cancer immunotherapy [8]. However, these models typically have limited longevity, are prone to graft-versus-host disease (GVHD), and lack the capacity for full immune system regeneration.

Our group has an established track record in performing PDX studies, particularly using combinations of targeted agents in HNSCC PDX models [9]. We have also shown that high-quality genomics data can result from a standard clinical workflow and is able to derive a comprehensive genomic profile accounting for gene mutations, expression, and fusions [10]. We were interested in exploring the autologous humanized immune system (HIS) model, derived from an individual’s own peripheral blood, as it offers compelling advantages for personalized immunological research urgently needed in HNSCC. By using self-derived immune cells, this approach circumvents histocompatibility barriers, enabling the study of immune responses in a fully autologous context, which is particularly valuable for patient-specific therapies such as cancer immunotherapy, adoptive T-cell transfer, or checkpoint blockade [11]. It allows for real-time modeling of immune–tumor interactions or autoimmune phenomena within the genetic and epigenetic landscape of the donor, thereby enhancing translational relevance. Moreover, because it does not require the time-intensive differentiation of hematopoietic stem cells, the autologous model could offer a more rapid platform for testing needed in patients with advanced disease, requiring timely therapeutic decisions. Despite limitations in durability and the risk of graft-versus-host-like responses in xenografts, the ability to preserve immune specificity and functional fidelity further enhances the autologous model as an attractive tool for personalized medicine. Therefore, the goal of this study was to assess the feasibility of establishing an autologous in vivo research model that can be used to develop and test future clinical therapies that can favorably impact patient outcomes.

## 2. Results

A total of 28 patients with HNSCC whose tumors were used for PDX establishment were contacted for study participation; 23 patients had adequate tumor engraftment, and their characteristics are shown in Supplementary Table S1 and summarized in Table 1. Most contacted patients declined to participate in the study: six patients expressed a lack of interest; eight patients declined participation after reading the consent form; four patients had passed away due to disease progression; four patients were lost to follow-up. A total of four patients consented to the apheresis process. Two patients failed screening because of a lack of adequate venous access; one patient did not have an engrafted PDX; one patient underwent apheresis.

### 2.1. Cell Product Information

A total of 380.8 mL of apheresis product was collected from the patient following mobilization with G-CSF and plerixafor. A total of 160 × 10^6^ CD34^+^ cells were enriched by positive selection with the CliniMACS device, which were cryopreserved at 8 × 10^6^ cells/vial in 20 vials and shipped to Champions Oncology for in vivo studies.

### 2.2. Humanization with HNSCC MPB-CD34^+^ Cells

**Figure 1:**
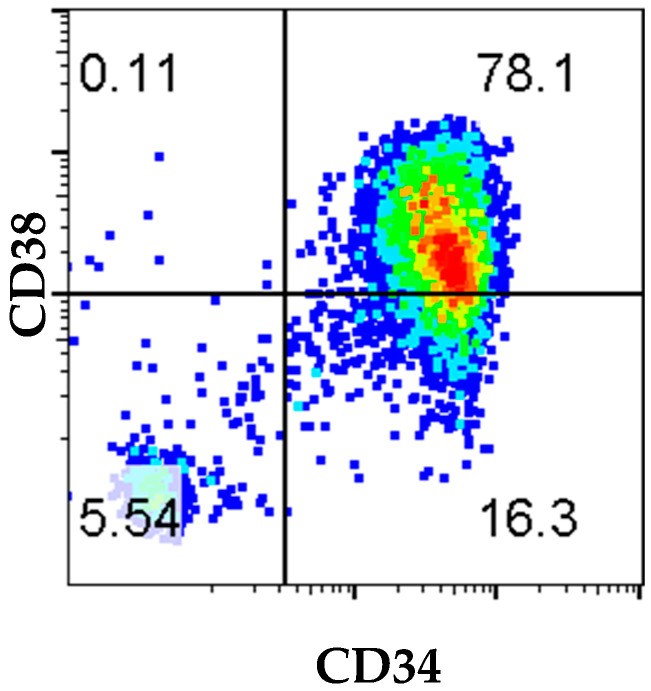
MPB-CD34^+^ day 0 phenotype. The purity of isolated cells from the mobilized peripheral blood (MPB) was assessed by flow cytometry, demonstrating a highly purified population of CD34^+^ hematopoietic stem cells (HSC). Assessment of primitive progenitors via flow cytometry for CD34 and CD38 indicated that CD34^+^CD38^−^ primitive cells were less frequent than the CD34^+^CD38^+^ population (16.3 vs. 78.1%).

**Figure 2:**
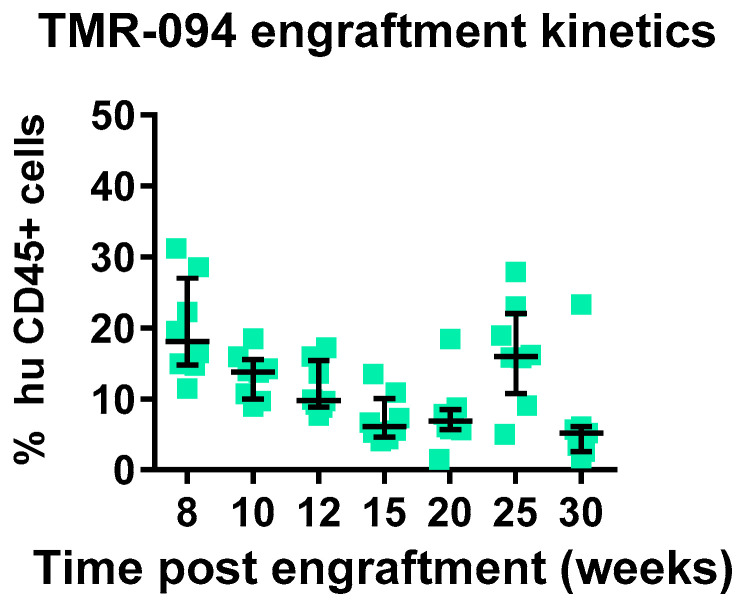
Human chimerism in NOG-EXL mice with MPB-CD34 cells. Engraftment of circulating human cells was observed from 8 weeks post-implant of 1 × 10^6^ MPB-CD34^+^ cells in irradiated NOG-EXL mice in the peripheral blood. Engraftment was assessed by comparing % human CD45 vs. % mouse CD45 in peripheral blood.

After thawing MPB-CD34^+^ cells from the HNSCC patient, and prior to in vivo transplantation, the phenotype and purity of MPB-CD34 cells were assessed by flow cytometry. Among live cells, 94.4% were CD34^+^, with 78.1% expressing CD34^+^CD38^+^ cells and 16.3% CD34^+^CD38^−^ cells (Figure 1). A total of 1 × 10^6^ cells were transplanted into irradiated 4–6-week-old female NOG-EXL (*n* = 8) mice post T-cell depletion treatment using UCHT1 antibody to prevent GVHD development from any residual T-cell presence post enrichment for CD34^+^ cells [12]. Engraftment (hCD45^+^) was monitored by flow cytometry by drawing peripheral blood samples for up to 25 weeks post-transplant. The highest percentage of circulating human CD45^+^ cells was observed in the periphery at 8 weeks after transplantation, 19.9%, with subsequent decline to 16.5% at 25 weeks and 6.8% at the 30-week terminal time point (Figure 2). These data demonstrated that MPB-CD34 cells derived from an adult HNSCC patient can engraft in NOG-EXL mice and stay stably engrafted for up to 25 weeks.

### 2.3. Immune Lineage Development in Periphery and Secondary Lymphoid Organs

**Figure 3:**
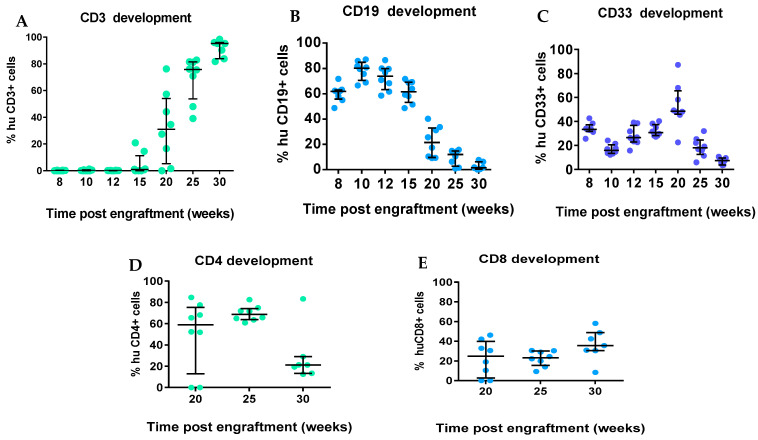
Immune lineage and T-cell subset development. Immune cell lineage development was observed in the peripheral blood post-CD34^+^ implantation in NOG-EXL mice. (**A**) CD3^+^ cell development had a delayed onset. (**B**,**C**) An inverse correlation was observed with CD19^+^ (**B**) and overall stable CD33^+^ cell development (**C**) with this adult MPB-CD34^+^ HSC in comparison to cord-blood-derived CD34 HIS development. (**D**,**E**) T-cell CD4 and CD8 subset development was observed post-engraftment in NOG-EXL mice.

**Figure 4:**
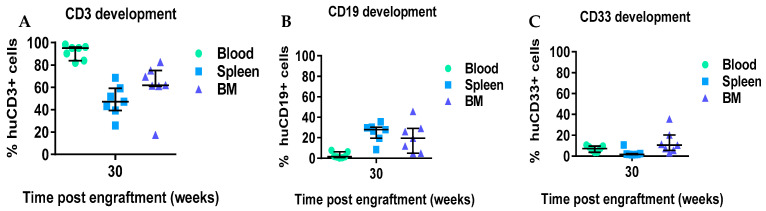
Immune cell subsets are detectable in blood, spleen, and bone marrow at 30 weeks. Immune subsets are detectable at varying levels in the blood, spleen, and organs. The highest frequency of CD3 was observed in comparison to CD19 and CD33. (**A**) T-cell levels. (**B**) B-cell levels. (**C**) Myeloid cell levels.

We also assessed the various immune lineages of human leukocytes up to 30 weeks post-transplant. Human CD3^+^ T cells in the blood of mice were observed at 15 weeks post-transplant (4.8% of human CD45^+^) and increased over time to a maximum of 90% CD3^+^ of huCD45^+^ by 30 weeks in periphery (Figure 3A). At 20 and 25 weeks, the composition of CD4:CD8 T cells in the peripheral blood was approximately 2:1 (50% to 34% of CD4^+^ and CD8^+^ T-cell subsets, respectively), with similar levels of both CD4 and CD8 T-cell subsets by week 30 (Figure 3D,E). High B-cell frequencies were observed at 8 (60.3%) and 10 (78.3%) weeks post-transplant of MPB-CD34 cells, with a continuous decrease observed until 25 weeks (9.7%) (Figure 3B). We also evaluated CD33 development since the presence of huIL-3 and GM-CSF expressed by NOG-EXL mice supports the development of myeloid cells. At early timepoints (8–15 weeks post-transplant), more CD33^+^ cells were observed among recipients of MPB-CD34 in comparison to T cells, but at later timepoints, CD33^+^ myeloid cells decreased (Figure 3C). Next, we investigated and compared the frequencies of immune cells in the blood, spleen, and bone marrow (BM). T cells were most plentiful in the blood in comparison to the spleen and BM (Figure 4A). Low frequencies of CD19^+^ B cells were detected in blood at week 30, while moderate levels (~25%) were found in the spleen and BM. (Figure 4B). Higher percentages of CD33 cells were observed in the BM (13.4%) in comparison to the blood and spleen (7% and 3%, respectively, Figure 4C). This data is quite limited, given that its application involved one patient only. Yet despite this significant limitation, taken together, the data demonstrate that human immune lineages, including myeloid cells, can develop from MBP-CD34 cells from adult HNSCC cancer patients and allow for engraftment and chimerism of immunodeficient mice with major immune compartments represented. The functionality of the immune lineages was not tested.

## 3. Discussion

In this current study, we have demonstrated the feasibility of human hematopoietic cell engraftment in NOG-EXL mice from 8–30 weeks following transplantation of MBP-CD34^+^ cells from an adult patient with HNSCC cancer. Our results provide a pilot model for the clinical and technical feasibility of successfully mobilizing CD34^+^ cells using the administration of G-CSF/Neupogen and plerixafor prior to apheresis on an IRB-approved protocol. Positive selection of CD34^+^ cells using the CliniMACS device resulted in an apheresis product with 94% of cells in the sample expressing CD34. The total number of enriched CD34 cell population recovered was sufficient to implant multiple mice, showing the feasibility of a successful study. Further analysis of HSC marker expression showed that only 16.3% of cells had the undifferentiated CD34^+^CD38^−^ phenotype, and 78% of cells were CD34^+^CD38^+^ (Figure 1). Evaluation of the immune reconstitution and surface phenotyping of cord blood (CB)-derived CD34 cells reported that CD34^+^CD38^−^ cells were responsible for long-term engraftment. However, both CD34^+^CD38^−^ and CD34^+^CD38^+^ subsets of CD34^+^ cells have been reported to home to BM and to differentiate and expand with different kinetics in expansion [13] The total number of cells and purity of CD34 cells can vary depending on patient status, age, and health, and can have a significant impact on questions that can be answered in an in vivo setting.

Significant limitations to study implementation primarily included the lack of participation of a significant number of patients in the apheresis process. Enrollment was further challenged by co-morbidities of patients with HNSCC, which were related to vascular disease and lack of vascular access, thus hindering our ability to enroll several patients. The presence of one analyzable patient significantly restricts the conclusive evidence as to the transplantation dynamics and immune recovery patterns. While this feasibility demonstration of establishing an autologous patient-derived xenograft (PDX) model in head and neck squamous cell carcinoma (HNSCC) is of value, its interpretation is limited by the reliance on a single patient. HNSCC is a biologically heterogeneous disease, and a single-patient model cannot capture the diversity of tumor behavior, immune interactions, or therapeutic response seen across the broader patient population. Additional limitations include the potential bias in PDX engraftment toward aggressive tumor clones and the technical variability inherent in the model. These limitations underscore the need for validation in larger, diverse cohorts to substantiate the translational relevance of this approach.

The observation of low CD34^+^CD38^−^ early progenitors coupled with decreased CD45^+^ cells at 25 weeks may highlight fundamental limitations in sustaining long-term immune reconstitution in the autologous model. CD34^+^CD38^−^ cells represent a primitive stem cell population capable of self-renewal and multilineage differentiation, critical for the continuous generation of immune cells, and scarcity in adult peripheral blood, especially when not supplemented with mobilized stem cells, implies a limited reservoir for ongoing hematopoiesis. Consequently, the declining CD45^+^ levels may indicate a lack of needed immune regenerative capacity. In contrast, cord-blood-derived HIS models, which are rich in CD34^+^CD38^−^ progenitors, may offer a more robust and sustained hematopoietic reconstitution, which in turn could be more facilitative to de novo T- and B-cell development and long-term multilineage output. However, as noted, a clear answer to this question in our model would require a larger sample size, which unfortunately was not possible in our study.

Another limitation of our study resides in the fact that the effectiveness of T-cell depletion could have influenced the composition and functionality of mature T-cell populations and potentially skewed the immune repertoire or reduced the diversity necessary for physiologically relevant responses. If T-cell removal is incomplete or uneven, residual mature T cells may dominate the immune landscape, leading to non-representative or artifact-prone immune activity. Conversely, overly aggressive depletion may impair engraftment or eliminate functionally important subsets. Compounding this challenge is the absence of functional immune assays, such as antigen-specific activation, cytokine production, or cytotoxicity tests, which prevents definitive assessment of the model’s immunocompetence. Without such assays, it is difficult to determine whether the engrafted immune cells can mount robust, antigen-specific responses, severely limiting the interpretability and translational relevance of findings derived from this model.

We assessed chimerism by assessment of circulating huCD45^+^cells in comparison to muCD45^+^ cells starting 8 weeks post-adaptive transfer of MPB-CD34 cells. In total, 100% of mice showed chimerism, with 20% huCD45^+^ cells circulating in the periphery, which was stable in circulation until 25 weeks, with a decline at the terminal 30 weeks to 6.8% (Figure 2). We also evaluated immune lineage development kinetics up to 30 weeks in the engrafted mice. An inverse correlation was observed between levels of T-cell development and B-cell development, whereas overall stable levels of CD33 were observed between 8 and 25 weeks post-transplant, with modest declines seen at the 30-week terminal timepoint. We and others have observed and reported a similar inverse correlation between T-cell development and B-cell development (data not reported). We observed a delayed onset of T-cell development starting at 15 weeks in this experiment using CD34^+^ cells from an adult HNSCC patient, whereas CB-derived CD34 cells support superior engraftment and T-cell development as early as 12 weeks after transplantation [14,15]. This difference could be potentially explained due to the presence of more CD34^+^CD38-ve cells. CD4^+^ and CD8^+^ T cells were observed in blood starting at 20 weeks and continuing to 30 weeks post-transplantation, with initially higher CD4:CD8 ratios at 20 and 25 weeks, with subsequent decreases in CD4^+^ T cells and increases in CD8^+^ T cells coincident with the development of xeno-GVHD at 30 weeks. We also evaluated the presence of immune cells in the spleen and BM at 30 weeks. Higher levels of CD3^+^ cells were detected in blood, spleen, and BM at 3 weeks in comparison to the numbers of CD19^+^ and CD33^+^ cells. Taken together the data suggest either (1) the CD34 hematopoietic stem cells derived from mobilized adult HNSCC are capable of engraftment and differentiation to T cells, or (2) that rare mature T cells contaminating the CD34^+^ cells selected from the mobilized apheresis product expanded slowly in the mice over the 30 weeks without causing xeno-GvHD for more than 6 months. In either case, these data suggest the feasibility of developing xenograft (PDX) comprised of autologous tumor immunografts using CD34^+^ cells and a tumor from the same patient co-engrafted, and allowing for therapeutic assessment of new therapeutic modalities.

Notably, the absence of a clear tissue-based confirmation of immune cell overactivity at 30 weeks restricts the ability to validate systemic immune persistence and function at the site of potential pathology or therapeutic relevance. While peripheral blood markers may suggest ongoing immune activity, without histological or immunophenotypic evidence from tissues, such as lymph nodes, spleen, or tumor microenvironments, it remains unclear whether engrafted immune cells maintain functional infiltration, activation, or effector responses over time. This gap hinders the interpretation of long-term immune dynamics and compromises the model’s utility for studying chronic immune processes, such as sustained tumor-immune interactions, autoimmunity, or immune-mediated toxicities.

Whether the relatively high proportion of CD33^+^ myeloid cells observed may reflect a biologically relevant parallel to the myeloid-rich tumor microenvironment commonly seen in HNSCC is unclear, yet possible. It is widely accepted that the exposure of the CD34^+^ cells to the human GM-CSF cytokine produced by the NOG-EXL mouse does result in the engraftment and development of CD33^+^ myeloid cells; however, it cannot be excluded that the HNSCC indication, which is often characterized by an abundance of immunosuppressive myeloid populations, including myeloid-derived suppressor cells (MDSCs) and tumor-associated macrophages (TAMs), plays a critical role in the presence and function of these CD33^+^ cells in immune evasion and therapeutic resistance. An autologous model enriched in CD33^+^ cells could thus offer a more faithful recapitulation of this immunosuppressive landscape, potentially enabling more accurate evaluation of immunotherapies targeting myeloid-driven resistance mechanisms. However, it remains essential to confirm whether these CD33^+^ cells functionally resemble their tumor-infiltrating counterparts, as mere phenotypic presence without validated suppressive activity may not fully capture the complexity of the HNSCC immune milieu.

To summarize, the significant limitations observed in establishing this autologous HNSCC model consist of challenges in the enrollment of HNSCC patients and in mobilizing sufficient CD34^+^ cells from a relatively elderly population. Alternative models using non-selected PBMCs, leading to a faster engraftment yet a greater potential for xeno-GVHD, are being evaluated [16]. In addition, new technologies such as 3D ex vivo assays are allowing a deeper understanding of the tumor immune compartment, therapeutic treatment interactions, and high-throughput assessment of different treatment options for patients. Despite our success in the establishment of autologous HIS-PDX mice with the potential to significantly advance translational immuno-oncology drug discovery and development, the practicality of peripheral autologous stem cell collection by apheresis remains a major obstacle to the widespread application of this modality. Exploration of alternative methods for the collection of CD34^+^ cells from adult cancer patients remains worthy of investigation.

## 4. Materials and Methods

### 4.1. Humanized Mouse Models

Champions Oncology has previously demonstrated the generation of the ImmunoGraft^TM^ platform, whereby two technologies, patient-derived xenograft (PDX) and humanized mice (immunodeficient mice reconstituted with a human immune system), are combined in a single platform (HIS mice). In this process, immune-compromised NOG (*Prkdc^scid^Il2rg^tm1Sug^*) mice are reconstituted with human CD34^+^ cells (typically from cord-blood) and monitored for the expansion of human immune cells (humanized). Humanized mice are engrafted with solid tumors, subjected to histocompatibility typing, and characterized by specific molecular markers, including PD-L1 expression [12].

Results from this program confirmed that mature human CD45^+^ cells comprised close to 50% of the leukocytes in the circulation and lymphoid organs of humanized mice. Solid tumors, including NSCLC, melanoma, and head and neck cancer, were successfully engrafted in humanized mice, and high expression of PD-L1 was found in approximately 80% of these tumors. When treated with anti-CTLA4 or anti-PD1 antibodies, systemic immune responses characterized by robust proliferation of splenic and circulating huCD3^+^ T cells, as well as activated huCD4^+^ Th1 cells, were noted. There was also an increase in tumor-infiltrating huCD8^+^ cytotoxic T lymphocytes and huCD68^+^ macrophages, along with elevated secretion of human-specific cytokines. Tumor growth inhibition, and in some instances, tumor regression, was demonstrated in treated HIS models, with the magnitude of growth inhibition correlating with the level of immune activation. Thus, the HIS platform is more reflective of the human tumor microenvironment and could provide a translationally relevant model for screening immune-therapeutic agents targeting the immune system [12].

In this study, we assessed the engraftment potential of adult MPB-CD34 cells from HNSCC cancer patients in the NOG-EXL mouse strain, which harbors human IL-3 and GM-CSF genes, allowing for superior engraftment and immune lineage development, including myeloid reconstitution [17]. We first irradiated sub-lethally NOG-EXL mice to improve overall human chimerism. This was followed by the adoptive transfer of 1 × 10^6^ 95% viable MPB-CD34 cells, which were pretreated with UCHT1 antibody to deplete any donor T cells remaining to prevent both acute and chronic GVHD [12].

### 4.2. Patient Selection and Enrollment

The mobilization stem cell protocol for patients who have engrafted HNSCC tumors in PDX models was approved by the Emory IRB. The plan was to enroll up to 10 patients with HNSCC over the course of one year.

Patients at least 18 years of age with local, locally advanced, or metastatic squamous cell carcinoma of the oropharynx, larynx, nasopharynx, hypopharynx, oral cavity, or sino-nasal squamous cell carcinomas who had previously provided tissue for PDX were eligible to enroll in the apheresis protocol. Eligible patients had to be free of any major medical condition that, in the assessment of the transplant physician, would place the donor at significant risk for serious or life-threatening complications. Enrolled patients required adequate antecubital veins or femoral veins to permit the collection of autologous stem cells by apheresis. None of the patients required a central line placement. Screening involved providing a written description of the study procedures to patients. Eligibility is needed to satisfy the criteria outlined in the standard operating procedure of the Winship Cancer Institute, “Related Donor Selection for Bone Marrow or Peripheral Blood Progenitor Cell Collection”. Enrolled subjects were examined by a transplant Advanced Practice Provider or transplant physician to determine suitability for collecting autologous stem cells using peripheral veins and to obtain informed consent for peripheral stem cell collection. Stem cell collection was followed by the administration and recording of the Autologous Donor History Questionnaire by the designated BMT Nurse Coordinator. Enrolled subjects underwent standard of care labs, 12-lead EKG, PA, and lateral chest radiograph as required for donor evaluation. Patients were excluded if they had any medical condition that, in the opinion of the investigator, would put the patient at risk during the mobilization or apheresis procedure.

### 4.3. Mobilization Procedure and Isolation of CD34^+^ Cells (MPB-CD34)

Enrolled patients received 5 daily injections of G-CSF at a dose of 10 µg/kg and, for patients with less than 20 CD34^+^ cells/µL in their blood on day 4 of G-CSF administration, a single s.c. injection of plerixafor at a dose of 0.24 mg/kg, followed by a standard 4 h or 24-L apheresis from their peripheral veins on day 5. The entire apheresis product was processed for CD34 enrichment using cliniMACS and cryopreserved overnight in a controlled-rate liquid nitrogen freezer. Enriched and cryopreserved PB-CD34 cells were shipped to Champions Oncology and were stored in a vapor phase until implantation into mice.

### 4.4. Mice and Engraftment

All animal studies were conducted under an IACUC-approved protocol in an AAALAC-certified facility and housed in microisolator cages. Euthanasia was performed, when necessary, based on IACUC-accepted protocols. NOD.Cg-*Prkdc^scid^ Il2rg^tm1Sug^* Tg (SV40/HTLV-IL3,CSF2)10-7Jic/JicTac (NOG-EXL) female mice aged 4–6 weeks were obtained from Taconic (Rensselaer, NY, USA). Mice were acclimatized for a week. On the day of implantation, mice were sub-lethally irradiated with 150 cGy whole body irradiation (RS 2000—X-ray Biological Irradiator, RadSource Technologies, Inc., Buford, GA 30518, USA). Within 4 h of irradiation, MPB-CD34^+^ cells (1 × 10^6^ cells) pretreated with UCHT1 antibody (BioXCell, Lebanon, NH 03766) by incubating the cells in suspension with UCHT1 antibody for 30 min on ice at a concentration of 1 µg/1 million WBC to deplete any donor T cells were then transplanted by intravenous injection (I.V.) (200 µL) in the tail vein. All mice were provided with acidified water and diet gel post-irradiation. Body weights were taken on days 0, 3, and 7 post-transplantation to monitor the recovery of mice. Mice were monitored weekly [12].

### 4.5. Flow Cytometry for Determination of Human Chimerism

Human cell chimerism and percentages of human cell populations were determined in peripheral blood from week 8 to 25 post-implant. An amount of 100 µL of blood was collected from individual mice via submandibular bleed in K_2_EDTA tubes, followed by immunostaining with fluorochrome-labeled antibodies. On week 30, terminal blood was collected by cardiac puncture in K_2_EDTA tubes, spleen and bone marrow were collected and processed for immunostaining with fluorochrome labelled antibodies to murine CD45, and antibodies to human CD45, CD3, CD4, CD8, CD33, CD34, CD38, and CD19 (Biolegend, San Diego, CA, USA). Stained and fixed cells were acquired on the MACSQuant^®^ Analyzer 10 (Miltenyi Biotec Inc, San Diego, CA, USA). Data analysis was performed using FlowJo V8 (FlowJo LLC, Ashland, OR, USA). After gating on the live, singlet leukocyte (FSC/SSC) population, the chimerism of mice was calculated based on the frequency of human CD45^+^ cells divided by total live cells (huCD45^+^ and muCD45^+^) × 100%. Data was plotted using GraphPad Prism, V6 (GraphPad software, San Diego, CA, USA).

## Figures and Tables

**Table 1:** Patient characteristics.

Characteristic		Number (%, N = 23)
Age	Mean (range)	58.7 (35–74)
	Not available	6 (26%)
Sex	Male	17 (73.9%)
	Female	4 (17.4%)
	Not available	2 (8.7%)
Ethnicity	Caucasian	5 (21.7%)
	Black/African American	4 (17.4%)
	Not available	14 (60.9%)
Tumor site	Larynx	7 (30.4%)
	Mouth	5 (21.7%)
	Mandible	2 (8.7%)
	Oral cavity	2 (8.7%)
	Sinus	2 (8.7%)
	Tongue	2 (8.7%)
	Pharynx	1 (4.3%)
	Buccal	1 (4.3%)
	Jaw (soft tissue)	1 (4.3%)
Disease stage	III	1 (4.3%)
	IV	20 (87.0%)
	Not available	2 (8.7%)
Tumor grade	Poorly differentiated	5 (21.7%)
	Poorly/moderately differentiated	4 (17.4%)
	Moderately differentiated	11 (47.8%)
	Well differentiated	1 (4.3%)
	Not available	2 (8.7%)
Primary vs. recurrent tumor	First diagnosis	13 (56.5%)
	Recurrent	9 (39.1%)
	Not available	1 (4.3%)
Treatment history	Naïve	13 (56.5%)
	Pretreated	7 (30.4%)
	Not available	3 (13.0%)
Previous radiation	Yes	6 (26.0%)
	Unknown	17 (73.9%)
Smoking history	Non-smoker	6 (26.0%)
	Former smoker	7 (30.4%)
	Smoker	8 (34.8%)
	Not available	2 (8.7%)

## Data Availability

Research data are not available currently.

## Supplementary Materials

The following supporting information can be downloaded at: https://www.mdpi.com/article/10.3390/ijms26115269/s1.

## Abbreviations

PDX	patient-derived xenograft
HIS	human immune system
MPB	mobilized peripheral blood
