# Supplementary material for: Feasibility Assessment of Autologous Human Immune System (HIS) ImmunoGraft Platform Development Using Autologous Mobilized Peripheral Blood (MPB) CD34 Cells Derived from Adult HNSCC Patient
Source: Int J Mol Sci. 2025 May 30;26(11):5269. doi: 10.3390/ijms26115269 (PMC12154748; doi:10.3390/ijms26115269)
Supplement: Supplementary file 1 [file ijms-26-05269-s001.zip › ijms-3605278-supplementary.pdf]

Supplemental Table S1: Individual patient characteristics

Tumor graft ID	Previous radiation	Harvest site	Tumor grade	Diagnosis	Treatment history	Disease stage	Smoking history	Age	Sex	Ethnicity
TMR-043A	Unknown	Mandible	Moderately differentiated	Recurrent	Naïve	IV	Former smoker	65	Male	N/A
TMR-044A	Yes	Mouth	Poorly differentiated	Recurrent	Pretreated	IV	Non-smoker	63	Male	N/A
TMR-049A	Unknown	Larynx	Poorly differentiated	First diagnosis	Naïve	IV	Former smoker	48	Female	N/A
TMR-051A	Unknown	Mouth	Moderately differentiated	First diagnosis	Naïve	IV	Smoker	69	Male	N/A
TMR-053A	Unknown	Mouth	Poorly/ moderately differentiated	First diagnosis	Naïve	IV	Non-smoker	44	Male	N/A
TMR-061A	Unknown	Oral cavity	Moderately differentiated	First diagnosis	Naïve	IV	Non-smoker	35	Male	Black or African American
TMR-068A	Yes	Oral cavity	Moderately differentiated	Recurrent	Pretreated	IV	Smoker	68	Male	Black or African American
TMR-086A	Unknown	Larynx	Moderately differentiated	Recurrent	Naïve	IV	Former smoker	58	Male	N/A
TMR-088A	Unknown	Larynx	Poorly differentiated	First diagnosis	Naïve	IV	Smoker	44	Male	Black or African American
TMR-089A	Yes	Larynx	Poorly differentiated	Recurrent	Pretreated	IV	Former smoker	69	Male	N/A
TMR-091A	Yes	Pharynx	Moderately differentiated	Recurrent	Pretreated	IV	Former smoker	58	Female	Caucasian
TMR-273A	Unknown	Mandible	Moderately differentiated	First diagnosis	Naïve	IV	Non-smoker	N/A	Male	N/A
TMR-052A	Unknown	Larynx	Moderately differentiated	First diagnosis	Naïve	IV	Smoker	54	Male	N/A
TMR-077A	Yes	Larynx	Poorly/ moderately differentiated	First diagnosis	Pretreated	IV	Smoker	53	Male	N/A
TMR-085A	Unknown	Sinus	Well differentiated	Recurrent	Naïve	IV	Smoker	74	Female	Caucasian
TMR-097A	Unknown	Sinus	Moderately differentiated	First diagnosis	Naïve	IV	Non-smoker	74	Male	N/A
TMR-094A	Yes	Mouth	Poorly differentiated	Recurrent	Pretreated	III	Former smoker	65	Male	Caucasian
TMR-308A	Unknown	Tongue	Moderately differentiated	First diagnosis	Naïve	N/A	Smoker	N/A	Male	N/A
TMR-318A	Unknown	Buccal	N/A	N/A	Pretreated	N/A	N/A	N/A	N/A	N/A
TMR-319A	Unknown	Jaw (soft tissue)	N/A	First diagnosis	N/A	IV	Non-smoker	57	N/A	N/A
TMR-331A	Unknown	Mouth	Poorly/ moderately differentiated	Recurrent	Naïve	IV	Former smoker	N/A	Female	Caucasian
TMR-350A	Unknown	Larynx	Poorly/ moderately differentiated	First diagnosis	N/A	IV	N/A	N/A	Male	Black or African American
TMR-371A	Unknown	Tongue	Moderately differentiated	First diagnosis	N/A	IV	Smoker	N/A	Male	Caucasian

N/A = not available
